# Utility of a Navigated High-Speed Drill in Robotic-Assisted Screw Placement for Spine Surgery

**DOI:** 10.7759/cureus.52779

**Published:** 2024-01-23

**Authors:** Makoto Ito, Jun Ueno, Yoshiaki Torii, Masahiro Iinuma, Atsuhiro Yoshida, Ken Tomochika, Takahiro Hideshima, Hisateru Niki, Tsutomu Akazawa

**Affiliations:** 1 Department of Orthopaedic Surgery, St. Marianna University School of Medicine, Kawasaki, JPN

**Keywords:** deviation rate, accuracy rate, navigated high-speed drill, skiving, robotic-assisted pedicle screw placement, spinal robotics system

## Abstract

Purpose

To elucidate the utility of a navigated high-speed drill used after the version upgrade in surgeries assisted by a spinal robotics system.

Methods

The subjects were 166 patients who underwent screw placement using a spinal robotics system between April 2021 to July 2023. A significant change during the study was the introduction of a navigated high-speed drill in 80 post-upgrade cases, aimed at improving drilling accuracy. Screw accuracy was analyzed using the Gertzbein and Robbins classification on postoperative CT scans. Screws placed before (pre-upgrade group: 718 screws in 86 cases) and after the system upgrade (post-upgrade group: 747 screws in 80 cases) were compared in terms of perfect accuracy and deviation rates.

Results

There were no significant differences in demographics or surgical details between the two groups. No significant differences were observed in the overall perfect accuracy rate and deviation rate (2.4% pre-upgrade vs. 2.0% post-upgrade) between the two groups. For the percutaneous pedicle screw (PPS), the perfect accuracy rate was significantly higher, and the deviation rate was significantly lower in the post-upgrade group (26.1% pre-upgrade vs. 4.4% post-upgrade). Notably, the post-upgrade group achieved 100% perfect accuracy and 0% deviation for the cortical bone trajectory screw (CBT) technique.

Conclusions

The introduction of the navigated high-speed drill did not significantly alter the overall perfect accuracy or deviation rates for robotic-assisted screw placement. However, its use did demonstrate improved outcomes in specific techniques such as PPS and CBT, indicating its potential value in addressing skiving in robotic-assisted minimally invasive surgeries.

## Introduction

Many studies have reported on the accuracy of pedicle screw placement using spinal robotics systems. Most of these studies use the Gertzbein and Robbins grade [1] as a standard, defining Grades A and B (deviations of less than 2 mm) as accuracy and calculating its precision. According to a systematic review in 2017, the accuracy of the early models (SpineAssist, Renaissance, ROSA robot) ranged from 85% to 100% [2]. Moreover, a meta-analysis reported in 2022 showed that the accuracy of the older generation models (SpineAssist, Renaissance) was 97%, while the newer generation models (Mazor X, Excelsius GPS, ROSA Spine, TINAVI) exhibited an accuracy of 99%, indicating that the newer models were more precise [3].

While robotic-assisted screw placement has been reported to have high accuracy, skiving has been pointed out as a primary factor for deviations [4]. Irregular bone surfaces, steep entry point angles, soft tissue pressure, high drilling pressure, and dull drill bits have been reported to induce skiving [5-9]. Therefore, attempts are being made to reduce skiving and improve screw accuracy through improvements to the drill, software, bone clamp, and outer cannula. As one of the methods to prevent skiving, the use of a high-speed drill has been recommended [10-13]. The high-speed drill with a squared drill bit is believed to prevent drill skiving due to its reduced likelihood of slipping on bone surfaces. In particular, the navigated high-speed drill is expected to further promote more accurate screw hole creation and contribute to the reduction in deviation rates.

The aim of this study is to elucidate the utility of the navigated high-speed drill used after the version upgrade in surgeries assisted by a spinal robotics system. It was hypothesized that the use of the navigated high-speed drill improved the accuracy of the screws.

## Materials and methods

Participants

The Institutional Review Board of St. Marianna University School of Medicine approved this retrospective study (approval No. 6188). The study involved 166 consecutive patients who underwent screw placement using the spinal robotics system (Mazor X Stealth Edition, Medtronic Inc., Dublin, Ireland) from April 2021 to July 2023. This comprised 66 males and 100 females, with an average age of 61.8 years (ranging from 12 to 89 years). The diagnoses included 109 with degenerative diseases (91 with lumbar spinal stenosis, eight with lumbar disc herniation, six with lumbar spondylolisthesis, and four with thoracic myelopathy), 34 with spinal deformities (24 with adolescent idiopathic scoliosis, seven with adult spinal deformity, two with syndromic scoliosis, and one with neuromuscular scoliosis), and 23 others (21 with vertebral fractures and two with metastatic spinal tumors).

Surgical workflow

All surgical procedures adhered to the "CT to Fluoro" workflow. Preoperative CT scans were obtained to meticulously plan screw placements. The spinal robotics system was programmed using these planning data. During surgery, we utilized a C-arm (STX-1000A from Toshiba Medical Systems, Ohtawara, Japan, or Zenition 70 from Philips, Amsterdam, Netherlands) to capture frontal and oblique X-ray images. These images were matched with our preoperative plans. Screw insertion, including drilling and tapping, was performed under the guidance of the robotic arm without using Kirschner-wire guidance. Nine surgeons performed pedicle screw placement. Six surgeons were board-certified spine surgeons, and three surgeons were training to acquire qualifications as board-certified spine surgeons.

**Figure 1:**
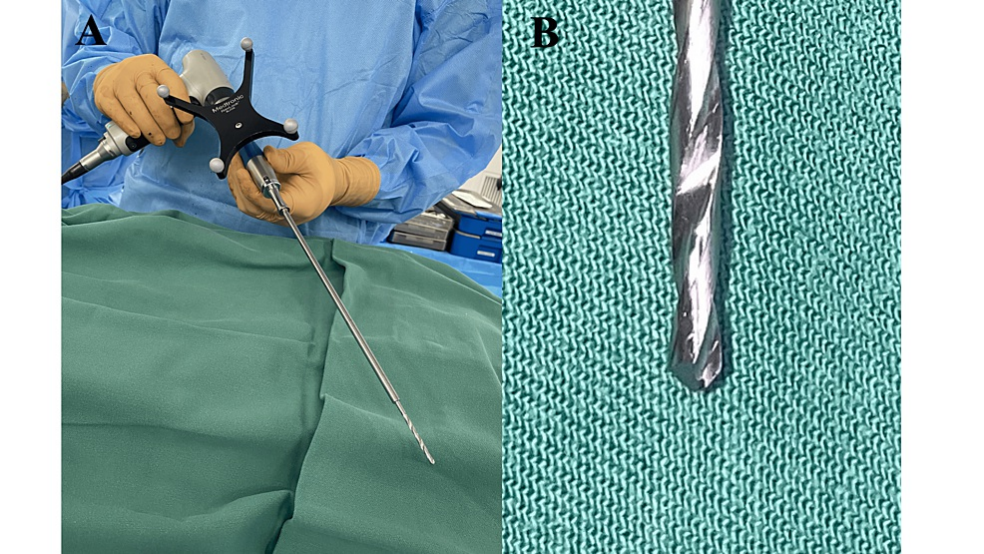
(A) In the 86 cases before the upgrade, a traditional drill was used. (B) A traditional drill bit was tapered.

During this clinical course, a major system upgrade took place. In the 86 cases before the upgrade, a traditional drill with a tapered bit was used (Figure 1). This traditional drill was used to create pilot holes on the bone surface. Direct observation sometimes revealed skiving, or slipping, especially on irregular or steep bone surfaces. Hence, in thoracic vertebrae, transverse processes were removed with rongeurs, or holes were created with a regular high-speed burr before using the traditional drill.

**Figure 2:**
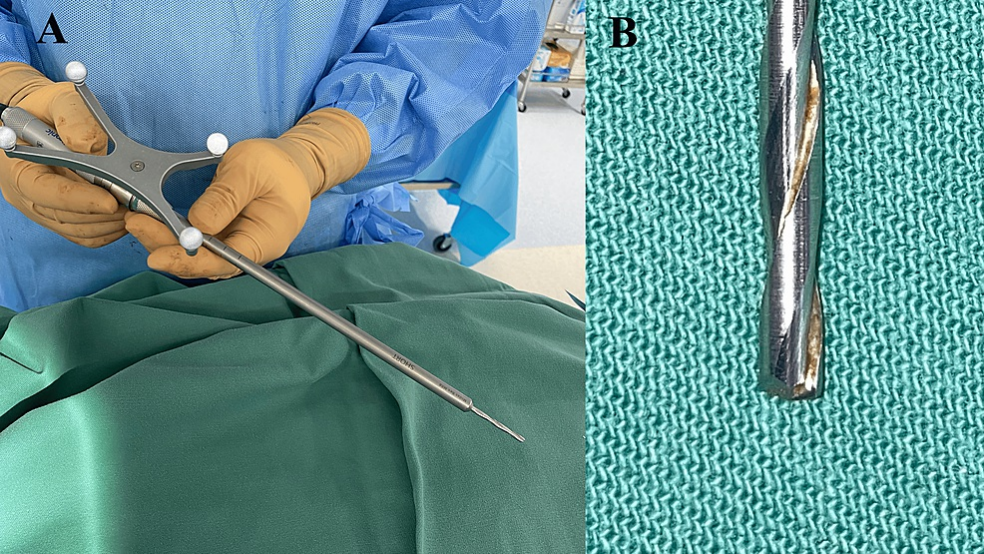
(A) In the 80 post-upgrade cases, the navigated high-speed drill with a square-shaped tip. (B) A high-speed drill has a square bit.

**Figure 3:**
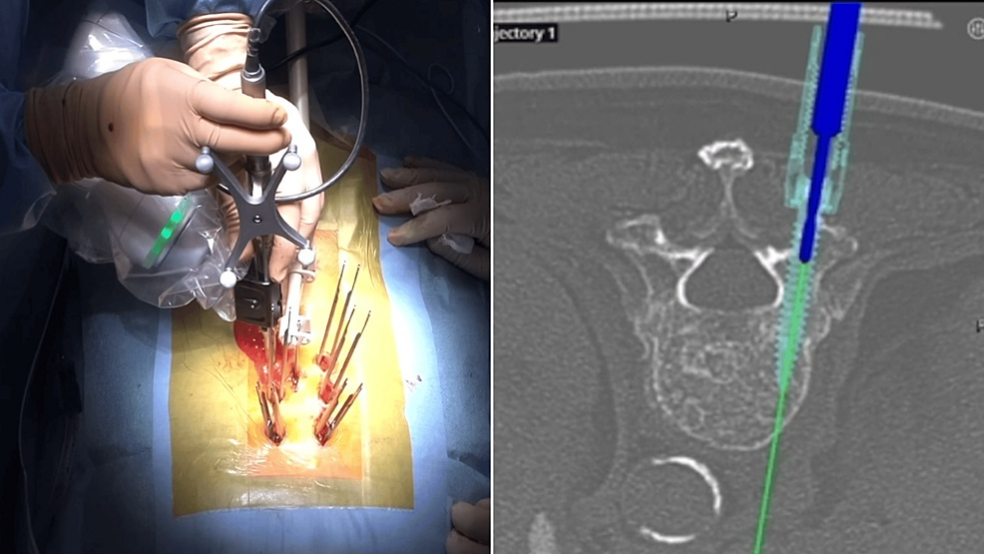
The high-speed drill can be viewed on the navigation screen.

In the 80 post-upgrade cases, the navigated high-speed drill (Mazor Midas, Medtronic Inc., Dublin, Ireland) was used under the guidance of the robotic arm (Figure 2). This is a high-speed drill with a square-shaped bit that can be viewed on the navigation screen (Figure 3). The high-speed drill was employed for creating pilot holes before using the traditional drill.

Evaluation

Screw Accuracy (Perfect Accuracy Rate and Deviation Rate) 

Postoperative CT scans were evaluated for screw deviations using the Gertzbein and Robbins classification [1]: Grade A: no breach of the cortical bone, Grade B: breaches <2 mm, Grade C: breaches of ≧2 mm but <4 mm, Grade D: breaches of ≧4 mm but <6 mm, and Grade E: breaches of ≧6 mm. Pedicle screw placement was assessed by one author (J.U.) who was blinded to the clinical symptoms. The perfect accuracy rate was defined as the proportion of Grade A, while the deviation rate was defined as the proportion of Grades C, D, and E (deviations ≧2mm) (Table 1).

**Table 1:** Gertzbein and Robbins classification and evaluation of screw accuracy. Source: [1]

Gertzbein and Robbins	
Grade A	No breach of the cortical bone
Grade B	Breaches <2 mm
Grade C	Breaches of ≧2 mm but <4 mm
Grade D	Breaches of ≧4 mm but <6 mm
Grade E	Breaches of ≧6 mm
Proportion of Grade A	Perfect accuracy rate
Proportion of Grades C, D, and E	Deviation rate

The 86 cases before the upgrade (718 screws) were categorized as the pre-upgrade group, while the 80 cases (747 screws) after the upgrade where the navigated high-speed drill became available were labeled the post-upgrade group. Perfect accuracy and deviation rates of screws were compared between these two groups.

Comparison of screw trajectory and insertion methods: Perfect accuracy and deviation rates of screws were compared between the two groups based on screw trajectory and insertion methods. The methods were classified into four types: traditional pedicle screw using open technique (Open-PS), cortical bone trajectory screw (CBT), percutaneous pedicle screw (PPS), and S2 alar iliac screw (S2AIS).

Comparison in vertebral levels: Perfect accuracy and deviation rates were compared across vertebral levels in both groups. Vertebral levels were classified into the following segments: first to fourth thoracic vertebra; T1-T4, fifth to eighth thoracic vertebra; T5-T8, ninth to 12th thoracic vertebra; T9-T12, first lumbar to first sacrum vertebra; L1-S1, and second sacrum vertebra to ilium; S2.

Statistical analysis

Normally distributed continuous variables were expressed as the mean ± standard deviation. For statistical analyses, Statistical Product and Service Solutions (SPSS, version 22.0; IBM SPSS Statistics for Windows, Armonk, NY) was used. Statistical analysis was performed using unpaired t-tests, chi-square tests, or Fisher's exact tests where appropriate, with p<0.05 considered statistically significant.

## Results

Demographic data are shown in Table 2. There were no significant differences between the two groups in terms of age, gender, body mass index, diagnosis, or number of fused segments. Operation time and intraoperative blood loss were also not significantly different.

**Table 2:** Demographics and surgical details of pre- and post-upgrade groups. Normally distributed continuous variables were expressed as the mean ± standard deviation.

	Pre-upgrade group	Post-upgrade group	p value
Age, years	61.9 ± 23.7	61.8 ± 22.5	0.965
Gender (male/female)	33/53	33/47	0.752
Body mass index	23.1 ± 4.7	23.2 ± 4.0	0.912
Diagnosis (degenerative disease/deformity/others)	56/16/14	53/18/9	0.585
Number of fused segments	3.6 ± 3.0	4.2 ± 3.3	0.273
Operation time, min	251.3 ± 81.7	241.0± 94.0	0.450
Intraoperative blood loss, ml	288.3 ± 316.6	264.2 ± 270.8	0.600

No significant difference was observed in the overall perfect accuracy rate between the two groups (pre-upgrade group: 91.9%, post-upgrade group: 91.2%, p=0.639). The overall deviation rate was also not significantly different (pre-upgrade group: 2.4%, post-upgrade group: 2.0%, p=0.722) (Table 3). In the post-upgrade group, both instances of Grade E deviation in PPS were lateral breaches of the lumbar pedicle.

**Table 3:** Comparison of the overall perfect accuracy rate and deviation rate between pre- and post-upgrade groups.

	Pre-upgrade group	Post-upgrade group	p
Total number of screws	718	747	
Gertzbein-Robbins grade			
A	660	681	
B	41	51	
C	16	12	
D	1	1	
E	0	2	
Perfect accuracy rate	91.9%	91.2%	0.639
Deviation rate	2.4%	2.0%	0.722

Comparison in screw trajectory and insertion methods

For Open-PS, the post-upgrade group had a significantly lower perfect accuracy rate, but no significant difference in deviation rate. In CBT, the post-upgrade group had a perfect accuracy rate of 100% and a deviation rate of 0%. For PPS, the perfect accuracy rate was significantly higher in the post-upgrade group, and the deviation rate was significantly lower. For S2AIS, there were no significant differences in perfect accuracy or deviation rates between the two groups (Table 4). In the post-upgrade group, both instances of grade E deviation in PPS occurred outside the pedicle of the lumbar spine. There was no significant difference in Grade E rates of PPS between the two groups (p=1.000).

**Table 4:** Comparison in Open-PS, CBT, PPS, and S2AIS (screw trajectory and insertion methods). Open-PS; traditional trajectory pedicle screw with open procedure, CBT; cortical bone trajectory screw, PPS; percutaneous pedicle screw, S2AIS; S2 alar iliac screw, GR; Gertzbein-Robbins.

	Pre-upgrade group	Post-upgrade group	p value
Open-PS			
Number of GR grade A/B/C/D/E	379/35/7/0/0	277/44/5/0/0	
Perfect accuracy rate	90.0%	84.5%	0.042
Deviation rate	1.7%	1.5%	1.000
CBT			
Number of GR grade A/B/C/D/E	258/4/4/0/0	174/0/0/0/0	
Perfect accuracy rate	97.0%	100%	0.025
Deviation rate	1.5%	0%	0.156
PPS			
Number of GR grade A/B/C/D/E	15/2/5/1/0	210/7/7/1/2	
Perfect accuracy rate	65.2%	92.5%	<0.001
Deviation rate	26.1%	4.4%	0.001
S2AIS			
Number of GR grade A/B/C/D/E	8/0/0/0/0	20/0/0/0/0	
Perfect accuracy rate	100%	100%	1.000
Deviation rate	0%	0%	1.000

Comparison of vertebral levels

For T1-T4, there were no significant differences in perfect accuracy or deviation rates between the two groups, although the deviation rate in the post-upgrade group improved to 5.4%. For T5-T8, the perfect accuracy rate was significantly lower in the post-upgrade group. Conversely, while there were no significant differences in the deviation rates between the two groups, it was lower in the post-upgrade group. For T9-T12, L1-S1, and S2, there were no significant differences in perfect accuracy or deviation rates between the two groups (Table 5).

**Table 5:** Comparison of vertebral levels. GR; Gertzbein-Robbins, T1-T4; 1st to 4th thoracic vertebra, T5-T8; 5th to 8th thoracic vertebra, T9-T12; 9th to 12th thoracic vertebra, L1-S1; 1st lumbar to 1st sacrum vertebra, S2; 2nd sacrum vertebra to ilium.

	Pre-upgrade group	Post-upgrade group	p
T1-T4			
Number of GR grade A/B/C/D/E	19/5/3/0/0	29/6/2/0/0	
Perfect accuracy rate	70.4%	78.4%	0.562
Deviation rate	11.1%	5.4%	0.642
T5-T8			
Number of GR grade A/B/C/D/E	56/7/4/0/0	42/19/1/0/0	
Perfect accuracy rate	83.6%	67.7%	0.041
Deviation rate	6.0%	1.6%	0.367
T9-T12			
Number of GR grade A/B/C/D/E	138/10/2/0/0	153/13/4/0/0	
Perfect accuracy rate	92.0%	90.0%	0.564
Deviation rate	1.3%	2.4%	0.688
L1-S1			
Number of GR grade A/B/C/D/E	439/19/7/1/0	435/13/5/1/2	
Perfect accuracy rate	94.2%	95.4%	0.460
Deviation rate	1.7%	1.8%	1.000
S2			
Number of GR grade A/B/C/D/E	8/0/0/0/0	20/0/0/0/0	
Perfect accuracy rate	100%	100%	1.000
Deviation rate	0%	0%	1.000

## Discussion

Comparing the pre-upgrade group to the post-upgrade group, no significant differences were noted in the overall perfect accuracy or deviation rates. In PPS, both the perfect accuracy rate and deviation rate improved significantly in the post-upgrade group. In CBT, the perfect accuracy rate improved significantly in the post-upgrade group. Although not statistically significant, the deviation rate also improved. The post-upgrade group achieved a perfect accuracy rate of 100% and a deviation rate of 0% with the high-speed drill. Thus, the navigated high-speed drill is especially useful for minimally invasive techniques such as PPS and CBT.

For the upper thoracic vertebrae (T1-T4), no significant differences were noted in the perfect accuracy or deviation rates. However, the deviation rate improved to 5.4% in the post-upgrade group. In mid-thoracic vertebrae (T5-T8), the perfect accuracy rate significantly deteriorated in the post-upgrade group, but the deviation rate improved, though not significantly. In Open-PS, the post-upgrade group had a significantly lower perfect accuracy rate, but no significant difference in deviation rate. This is presumed to be due to the higher number of Grade B cases in T5-T8 and Open-PS in the post-upgrade group among those with scoliosis. In cases with scoliosis, the pedicle channels of the thoracic spine are often narrow. Therefore, to enhance the overall accuracy of Open-PS, it is believed that, even when using robotics, it is advisable not to place screws in the narrow pedicle of the thoracic spine. Even though the perfect accuracy rate has deteriorated, the deviation rate has not worsened despite an increase in cases within the acceptable range of Grade B, which is believed to pose no significant issues. For T9-T12, L1-S1, and S2AIS, no significant differences were noted in perfect accuracy or deviation rates.

In the post-upgrade group, both instances of Grade E deviation in PPS were lateral breaches of the lumbar pedicle. It is presumed that this displacement resulted from excessive pressure applied to the outer cannula, causing it to shift to the outer aspect of the lumbar facet joint. Since the displacement of the outer cannula is not directly visible during PPS, caution should be exercised to avoid excessive pressure on the bone surface while positioning the outer cannula.

While the screw deviation rate with robotics is reported to be as low as 2.2%, ensuring high safety, the deviation rate for robotic-assisted PPS is a concerning 10.3% [13]. Katsevman et al. reported that the deviation rate for the robotic-assisted PPS is 15.4%, not much different from that of the fluoroscopic-assisted PPS [14]. Since the PPS insertion point is not directly visible, it is hard to notice any skiving of the drill. When drilling pilot holes, if the drill meets an inclined bone surface, lateral skiving forces can misalign the drill, or the tool can bend and go off course. Using the high-speed drill with low propulsion can mitigate skiving [10]. In our study, the pre-upgrade group, which used a drill rotating at 120-180 rpm, had a high deviation rate of 26.1%. Meanwhile, the post-upgrade group, with a navigated high-speed drill rotating at 60,000 rpm, had a much lower rate of 4.4%.

For cervical pedicle screw insertion under navigation guidance, instead of using the traditional drill or probe, which might require significant downward pressure to penetrate the cortical bone, it is recommended to use a high-speed drill [15]. The high-speed drill can operate with minimal force and can effectively drill even into hard cortical bones. Thus, the anatomical structure of the spine and the position of the vertebrae remain unchanged during drilling, ensuring the precision of the navigation. Skiving is more likely on irregular bone surfaces or during insertion at steep angles to the bone surface [9]. A high-speed drill with a squared bit can prevent drill skiving, as it is less likely to slip on bone surfaces [10]. As a method to prevent skiving, the use of high-speed drills is recommended in robotic-assisted pedicle screw placement [11-13]. In our study, the squared-tipped navigated high-speed drill contributed to preventing screw deviations in minimally invasive techniques such as PPS and CBT.

There were several limitations to this study. Since it was not a randomized study, the number of cases compared and the number of screws were not the same between the two groups. Notably, in the pre-upgrade group, there were only 23 PPSs. This was because, before using the navigated high-speed drill, the deviation rate was perceived to be high, limiting its use in many cases of robotic-assisted PPS. The study included cases from the early adoption phase of robotic-assisted spine surgery. After the version upgrade, there could have been improvements in PPS and CBT accuracy rates due to increased proficiency with robot-assisted spine surgery. System upgrades included software improvements, changes to bone clamps, alterations to the outer cannula shape, and the inclusion of the navigated high-speed drill, so it cannot be said for certain that the navigated high-speed drill alone improved the deviation rates post-upgrade. The outer cannula shape can impact the stability of the interface between the outer cannula and the bone surface at the screw insertion site, and this stability may influence the deviation rate of pedicle screw placement. Although we believe that the inclusion of the navigated high-speed drill was the most significant improvement, there is also a possibility that the outer cannula contributed to the improvement in accuracy by having a thinner tip that is less susceptible to muscle pressure. Despite an improvement in the deviation rate of PPS, it remains slightly high. The high-speed drill proved effective in enhancing the PPS deviation rate, but this study did not explore further methods to reduce the deviation rate. We believe that addressing this issue should be a focus of future research endeavors.

## Conclusions

Whether the navigated high-speed drill was used or not, there were no significant differences in the overall perfect accuracy or deviation rates. The use of the navigated high-speed drill resulted in the observed improvements in the PPS deviation rate. In CBT, although there were no significant differences between the two groups, the post-upgrade group had a perfect accuracy rate of 100% and a deviation rate of 0%. This suggests that the navigated high-speed drill is valuable in addressing the skiving issues in robotic-assisted minimally invasive techniques.
